# AttentionDriveNet: Fusion of deep cognitive network with Attention modeling for robust navigation in Self-driving vehicles

**DOI:** 10.1371/journal.pone.0330933

**Published:** 2025-12-04

**Authors:** Sushruta Mishra, Rishabh Mohata, Hrudaya Kumar Tripathy, Jnyana Ranjan Mohanty, Kshira Sagar Sahoo, N. Z. Jhanjhi, Abdullah Alourani

**Affiliations:** 1 School of Computer Engineering, Kalinga Institute of Industrial Technology (Deemed to be University), Bhubaneswar, Odisha, India; 2 School of Computer Application, Kalinga Institute of Industrial Technology (Deemed to be University), Bhubaneswar, Odisha, India; 3 Department of Computer Science and Engineering, SRM University-AP, Amaravati, Andhra Pradesh, India; 4 School of Computer Science, SCS Taylor’s University, Subang Jaya, Malaysia; 5 Office of Research and Development, Asia University, Taichung, Taiwan; 6 Department of Management Information Systems, College of Business and Economics, Qassim University, Buraydah, Saudi Arabia; Gachon University, KOREA, REPUBLIC OF

## Abstract

Self-driving vehicles are envisioned as automated and safety-focused vehicles facilitating smooth movement on roads. This research proposes a novel, robust, and intelligent navigation framework for such vehicles through an integrated fusion of advanced technologies like predictive analytics with remote sensing and detection for accurate obstacle/object detection. TaskTrek, ViewVerse, and RuleRise form the core of the essential model governing vehicle-environment interaction. TaskTrek handles kinematic trajectory synthesis and space-time traffic modeling, ViewVerse provides LiDAR-based volumetric perception and radar-assisted navigational intelligence, and RuleRise manages topological localization, vehicle actuation, and autonomous decision-making through multimodal sensory fusion. The model applies an iterative Multi-FacBiNet method, which uses the cognitive Fully Convolutional Neural Network (FCNN) method to detect and classify obstacles during vehicle movement on the road. Upon stimulation during vehicle movement, the model provided an encouraging outcome. The fusion of predictive intelligence, Radar, and sensing technologies gave 95.3% proficiency. Minimum obstacle detection, processing, and response delays of 0.116 seconds, 0.105 seconds, and 0.36 seconds, respectively, are recorded. The computed mean obstacle detection accuracy for right, left, front and back camera angles are 88.3%, 83.8%, 91.4%, and 89.9%, respectively. Further, a comprehensive analysis of the model’s performance in different on-road scenarios considering metrics like traffic load, road type, and region density was done. The model generated a very impressive accuracy of obstacle detection on all parameters. The results of this study not only aid in accelerating the development of precise navigation-enabled self-driving vehicles but also in the context of environmentally friendly mobility/motion tracking solutions.

## 1. Introduction

The rise of autonomous vehicles (AVs) marks a transformative shift in modern transportation, driven by technological innovation. Equipped with artificial intelligence (AI) and sophisticated sensor systems, these vehicles can navigate and make real-time decisions without human intervention, redefining the conventional paradigm of human-controlled driving. At the core of autonomous driving are highly intricate technological systems that enable AVs to perceive, process, and interact with their surroundings effectively [1].

The benefits of AV technology extend beyond convenience; they enhance road safety, optimize fuel efficiency, and improve mobility for individuals who may be unable to drive [2]. By leveraging advanced sensor technologies such as LiDAR, radar, and cameras, these vehicles continuously gather real-time environmental data to minimize human errors and reduce accident rates. Furthermore, AI-driven decision-making systems allow AVs to respond instantly to complex and high-risk scenarios, ensuring both efficiency and safety in navigation. While the technical advancements in AVs are groundbreaking, societal challenges and ethical dilemmas remain central to their widespread adoption. Questions concerning liability in accidents, ethical decision-making in critical situations, and workforce displacement highlight the need for responsible integration of autonomous technology. This study examines not only the engineering mechanisms behind AVs but also the ethical concerns that arise when these machines make autonomous decisions in morally ambiguous situations [3].

Autonomous driving is structured into five levels of automation, ranging from Level 0 (complete human control) to Level 5 (full autonomy, requiring no human intervention). As automation progresses, the role of AI-driven perception, mapping, and decision-making becomes more critical. AV navigation relies on three fundamental components:

- Localization: Accurately determining the vehicle’s position in its environment, crucial for safe and precise navigation.
- Mapping: Constructing and maintaining an up-to-date digital representation of the surroundings, enabling real-time decision-making.
- Object Tracking: Continuously monitoring the movements of nearby vehicles, pedestrians, and obstacles to maintain situational awareness and proactive response.

The synergy between these three elements forms the foundation of reliable and efficient autonomous navigation. By integrating AI-driven perception and decision-making frameworks, AVs can enhance road safety, improve traffic efficiency, and adapt to dynamic environments. This paper explores the technological foundations, challenges, and ethical considerations in autonomous vehicle development, providing a comprehensive understanding of their evolving role in modern transportation.

**Fig 1:**
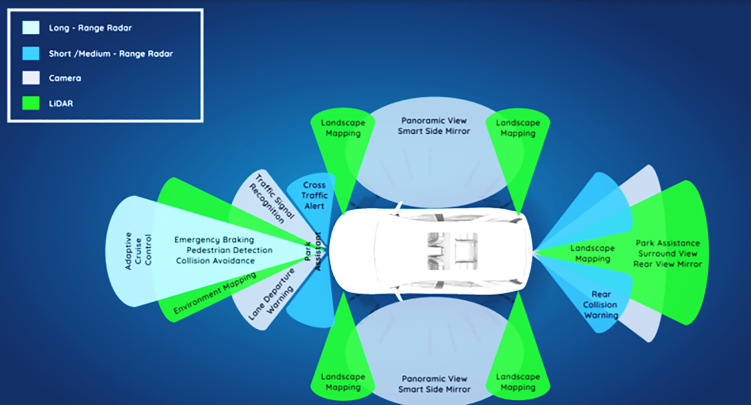
Sensory intelligence components in self-driving cars.

An automobile with detectors added for improved situational awareness and smart driving is apparent in Fig 1 underneath. The integration of multiple sensor technologies that are necessary for self-driving systems is depicted in the image. LiDAR allows for accurate 3D mapping of the environment and highly accurate tracking of lane markings and obstacles. Radar assists with speed estimation and object tracking, particularly in bad weather. High-resolution visual data from cameras are employed for lane detection, pedestrian tracking, and traffic sign recognition. Parking as well as low-speed movements are made easier with ultrasonic sensors, which assist close-range object identification. Every sensor has a specific function, but together they add to the car’s overall environmental awareness and enable optimum autonomous driving.

### 1.1. Problem statement

Autonomous vehicle navigation requires precise perception, real-time localization, and adaptive decision-making in dynamic environments. [4] Existing studies emphasize distributed computing in Internet of Vehicles (IoV) but often lack adaptability to high-speed urban scenarios, where latency, sensor fusion inconsistencies, and environmental uncertainty compromise decision accuracy. Traditional localization methods also struggle with occlusions, multi-sensor calibration errors, and real-time environmental adaptation, limiting their practical application. The limitations of current object detection algorithms further exacerbate these challenges. While these models demonstrate significant potential, they often fail to accurately discern interconnected objects in complex environments. A real-time object capturing and validation tool is essential to enhance spatial awareness, improve decision-making, and refine downstream computational processes. However, trade-offs persist across available models—some lack computational efficiency, others compromise accuracy, and none are fully optimized for challenging terrains. A critical example is Tesla’s Full Self-Driving (FSD) system, which has faced difficulties in handling unpredictable pedestrian behavior and occluded objects, leading to safety concerns. Similarly, Waymo’s self-driving taxis, despite operating successfully in controlled urban environments, struggle in unstructured settings where road anomalies and irregular traffic flows disrupt their decision models. These cases highlight the need for a comprehensive approach that integrates perception, prediction, and planning in real-world conditions. The challenge is particularly amplified in India, where rural roads constitute nearly 70% of the total road network. Outdated infrastructure, delayed construction, pedestrian interferences, and diverse road conditions present obstacles that conventional navigation models fail to address effectively. India’s traffic patterns, characterized by unregulated pedestrian movement and inconsistent lane discipline, necessitate an adaptive, high-precision navigation system designed for unstructured and semi-structured environments. To address these gaps, we propose a multi-layered framework integrating TaskTrek, ViewVerse, and RuleRise—three subsystems that enhance trajectory optimization, real-time object detection, and multimodal sensory fusion for autonomous decision-making. By taking advanced detection systems, spatiotemporal traffic heuristics, and computational intelligence, this framework fortifies the reliability, responsiveness, and situational awareness of autonomous vehicles, bringing real-time navigation to the forefront of human-inspired technological evolution.

This research integrates TaskTrek, ViewVerse, and RuleRise to provide an intelligent braking and navigation framework for self-driving cars. It offers smooth coordination between task execution and laws, improves selection with rule-based compliance, and increases brake efficiency. Using AI-driven planning and real-time sensor data, the model improves navigation while assessing performance in a variety of scenarios for usage in practice.

The main contributions of the work are as follows:

- This research designs a reliable navigation framework ‘AttentionDriveNet’ for self-driving vehicles that embeds an advanced fusion of deep neural networks with attention modeling, taking it beyond traditional frameworks.
- Radars, LiDars, and navigation algorithms are among the high-tech sensors that the model interacts with extensively and assist in obstacle recognition during vehicular movement.
- Multi-FacBiNet method maximizes neural network attention modeling in the AttentionDriveNet design with the use of Fully convolution neural network (FCNN), promoting cognitive functionality and focus. It raises durability and productivity by dynamically allocating attention to key portions in the data. With its emancipation the system can handle complex events with greater precision and speed.
- Implementation showed promising results from the model. When the two approaches were integrated, object identification performance was very high with low latency. Additionally, it recorded the best accuracy at detecting impediments at different camera angles while in operation. Also, the model was performed for a variety of on-road environments, and the results are very promising. The findings of this study contribute to the faster development of detailed, navigation-enabled autonomous cars.
- Thus, the model can accurately detect obstacles during vehicular navigation thereby enabling precise decision making for self driving vehicles.

## 2. Literature survey

The growing significance of Advanced Driver Assistance Systems (ADAS) in enhancing road safety through sensor-based technologies has been extensively explored. Sensor fusion has demonstrated substantial potential in advancing autonomous mobility solutions [1]. One innovative approach involves an image-based vehicle tracking system that utilizes stereo vision, image point fusion, and radar data integration to estimate position, exposure, and motion state, enabling robust tracking and path prediction from a moving platform [2]. Furthermore, integrating Microsoft Kinect depth data with 3D ray range scanner information has shown promise in real-time mobile systems, improving obstacle detection and road surface mapping operations [3].

The integration of Lane Keeping System (LKS) and Adaptive Cruise Control (ACC) into an LKS-ACC system provides novel lane detection techniques, overcoming occlusions and disturbances from neighboring vehicles [4]. The evolution of driver assistance systems highlights the transition from proprioceptive to exteroceptive sensors, emphasizing the importance of intermediate steps toward automated and cooperative driving, particularly in urban traffic environments [5]. Another approach explore the applications of Unmanned Aerial Vehicles (UAVs) in smart cities, emphasizing their role in surveillance, traffic monitoring, and disaster management. They highlight key challenges such as security threats, data integrity, and privacy concerns, proposing blockchain as a robust solution to enhance secure communication, data authentication, and decentralized control in UAV networks [6]. Mazhar et al. propose a symmetrically designed traffic engineering model for next-generation Wireless Sensor Networks (WSNs) that ensures Quality of Service (QoS) by employing a dynamic queuing mechanism. This mechanism assigns priorities based on input data type and queue length, aiming to reduce resource over-provisioning, delay, and packet loss ratio, thereby enhancing QoS efficiency in limited bandwidth networks with real-time communication [7]. Deep learning techniques in autonomous vehicle control have been categorized into lateral, longitudinal, and combined lateral-longitudinal control, addressing associated challenges [8]. The challenge of developing cost-effective ADAS solutions is recognized, underscoring the need for wider accessibility of ADAS technology to drivers [9]. Additionally, a smart vehicle over-speed detection system leveraging GPS, IoT, and sensors has been proposed to detect and report over-speeding vehicles, although it lacks real-time obstacle avoidance capabilities [10].

Research has also explored asset robots equipped with LABVIEW and CCD cameras, enabling remote control and wireless communication for accessing restricted areas efficiently [11]. Vehicle detection in computer vision remains a challenging task, prompting the development of a multi-scale vehicle detection system to improve performance, adapt to varying vehicle sizes, and address training imbalances [12]. A robust deep learning approach for detecting and identifying driver distraction has been introduced, employing dashboard-mounted cameras and convolutional neural network architectures with genetic algorithm-based optimization [13, 14]. Investigate the integration of electric vehicle (EV) charging systems within the smart grid, leveraging various machine learning techniques to optimize charging efficiency, load management, and energy distribution. Their study highlights the potential of intelligent algorithms in enhancing grid stability, reducing peak loads, and improving overall energy utilization in EV infrastructure. Object detection improvements have been driven by advancements such as Fast R-CNN, which leverages deep convolutional networks to achieve faster training, testing times, and improved accuracy [15]. The paper discusses the challenges of delivering seamless immersive experiences in autonomous vehicles, emphasizing network variability, computational constraints, and real-time processing. It further explores potential solutions, such as edge computing and AI-driven optimization, to enhance Quality of Experience (QoE) in these environments. Additionally, it highlights the need for standardized frameworks to ensure interoperability and efficient resource management [16].

Hybrid deep learning architectures have also been explored for autonomous vehicle perception. A notable study leverages the Hybrid YOLOv3 framework to propose a real-time multi-task scheme for vehicle and pedestrian tracking. This approach employs a single neural network for both tasks, reducing computational overhead while enhancing detection accuracy [17]. Using the UA-DETRAC benchmark dataset, the proposed model demonstrates superior tracking efficiency and precision through a combination of CNN-based tracking, Kalman filtering, and YOLOv3 object detection. However, its focus on vehicle recognition and tracking limits its adaptability to other event categories, restricting its generalization [18].

Another study presents a novel hybrid CNN-LSTM model for object detection in autonomous vehicles, integrating a convolutional neural network for feature extraction with a long short-term memory (LSTM) network for sequence modeling [19]. Khan et al. propose NPBMT, a novel buffer management technique designed to enhance data transmission efficiency in Internet of Vehicle-based Delay Tolerant Networks (DTNs). The study addresses key challenges such as network congestion and packet loss, demonstrating how the proposed approach optimizes data delivery and resource utilization in dynamic vehicular environments [20]. Additionally, an optimized algorithm incorporating neural architecture search, a dedicated small object detection layer, coordinate attention mechanisms, and structural re-parameterization enhances both speed and accuracy [21].

Our work builds upon these advancements by leveraging attention-driven architectures to improve object detection, obstacle avoidance, and trajectory planning in autonomous navigation. [22] Inspired by methodologies like Efficient Deep-fake Detection via Layer-Frozen Assisted Dual Attention Network and Visionary Vigilance: Optimized YOLOv8 for Fallen Person Detection, our model ensures real-time responsiveness in dynamic traffic scenarios through superior feature extraction, enhanced localization, and precise computational modeling.

This study integrates machine learning-based detection and IoT-enabled sensors to enhance accuracy in vehicle tracking, emphasizing the importance of real-time data processing in developing intelligent transportation systems for smart cities. The proposed approach improves traffic monitoring efficiency, enabling better decision-making and resource allocation for urban mobility solutions [23]. Adaptive Cruise Control (ACC) extends this functionality by dynamically adjusting speed to maintain safe distances from leading vehicles [24]. However, existing systems struggle to differentiate between straight and curved road segments, necessitating manual speed adjustments. Our proposed model addresses this limitation by comprehensively perceiving road conditions, including straight stretches, curves, rugged terrains, and potholes. Through advanced imaging sensors and a sensor fusion module integrating GPS data and a digital road map with speed limits, our system ensures precise environmental awareness and real-time adaptive speed control. Another analysis in [25] discusses a vehicular automated coordination model using advanced computing and cognitive learning to facilitate flexible navigation.

While some models rely on laser-based object and image detection [26], they often face challenges in effectively handling dynamic and rapidly changing objects, limiting their efficiency in real-time driver assistance tasks. In contrast, our approach introduces deep learning techniques for dynamic object recognition, ensuring significantly improved quality, clarity, and accuracy in real-time perception. By addressing existing gaps in the literature, our model enhances autonomous navigation and contributes toward safer, more adaptive driving solutions tailored to diverse and unpredictable environments. An overview of the existing models with its associated research gaps are summarized in Table 1.

**Table 1:** Overview of existing works and its related research gaps.

Author(s)	Contribution	Research Gap
Ziebinski et al. [1]	Conducted a survey on ADAS technologies and sensor fusion for autonomous mobility.	Lack of cost-effective ADAS solutions for wider accessibility.
Barth et al. [2]	Developed an image-based vehicle tracking system using stereo vision, image point fusion, and radar integration.	Need for improved robustness in dynamic real-time environments.
Nastjuk et al. [3]	Integrated Microsoft Kinect depth data with a 3D ray range scanner for obstacle detection.	Limited application in complex urban scenarios.
Ullah et al. [4]	Proposed an LKS-ACC system for lane detection and adaptive cruise control, overcoming occlusions.	Struggles in differentiating between straight and curved road segments.
Bengler et al. [5]	Explored the evolution of driver assistance systems from proprioceptive to exteroceptive sensors.	Need for intermediate steps to full automation in urban environments.
Shah et al. [6]	Investigated UAV applications in smart cities with blockchain for security and data authentication.	Challenges in security threats, data integrity, and privacy concerns.
Mazhar et al. [7]	Developed a traffic engineering model for QoS in Wireless Sensor Networks (WSN).	Resource over-provisioning and delay issues in real-time communication.
Kuutti et al. [8]	Categorized deep learning techniques in autonomous vehicle control.	Difficulty in handling real-time computational constraints.
Chaudhari et al. [9]	Discussed cost-effective ADAS solutions.	Existing systems are expensive, limiting accessibility.
Patel et al. [10]	Designed a smart vehicle over-speed detection system using GPS, IoT, and sensors.	Lacks real-time obstacle avoidance capabilities.
Singh et al. [11]	Developed remote-controlled asset robots using LABVIEW and CCD cameras.	Limited efficiency in dynamic and restricted environments.
Kumar et al. [12]	Created a multi-scale vehicle detection system for computer vision applications.	Struggles with varying vehicle sizes and training imbalances.
Mehta et al. [13]	Proposed a deep learning model for driver distraction detection using CNNs.	Optimization needed for real-time deployment in vehicles.
Sharma et et al. [14]	Designed a machine learning-based EV charging system for grid optimization.	Energy utilization and peak load reduction still need enhancement.
Gupta et al. [15]	Implemented Fast R-CNN for object detection improvements.	Computational constraints in real-time applications.
Verma et al. [16]	Explored QoE in autonomous vehicles using AI and edge computing.	Lack of standardized frameworks for interoperability.
Yadav et al. [17]	Developed a hybrid YOLOv3 model for vehicle and pedestrian tracking.	Limited adaptability beyond vehicle recognition tasks.
Rao et al. [18]	Introduced a CNN-LSTM model for object detection in autonomous vehicles.	Needs improvement in generalizability across different scenarios.
Khan et al. [19]	Proposed an NPBMT buffer management system for IoV-based DTNs to reduce congestion.	Still faces packet loss issues under high traffic loads.
Desai et al. [20]	Optimized a deep learning algorithm for object detection.	Needs improvement in detecting small objects in dynamic environments.

Though autonomous navigation has moved forward substantially the majority of current frameworks (such as Hybrid YOLOv3, CenterNet, and SSD) only focus on speed or detection accuracy, often achieving both under unpredictable and real-world road situations. Work on object detection or ADAS systems (such as Fast R-CNN and hybrid CNN-LSTM) tend to succeed in organized metropolitan settings and are not as flexible in unstructured areas like rural Indian roadways. Additionally, sensor fusion techniques mostly stay the same, not being able to deal with real-time occlusions and sensor noise in severe weather conditions.

Furthermore, cognitive mechanisms for attention are not suitably integrated into FCNNs for contextual prioritization of object detection in prior models. In convoluted intersections, this results in less accurate lane detection, delayed braking decisions, and less sharpness. By bringing together attention-driven deep learning (AttentionDriveNet), real-time perception (ViewVerse), trajectory optimization (TaskTrek), and legal-rule adherence (RuleRise), the approach we propose overcomes these drawbacks and shows enhanced performance in a variety of scenarios, such as busy cities, zigzag roads, and rural areas.

## 3. Materials and methodology

### 3.1. System requirements

A thorough integration of the hardware and software components is required for the system to be deployed successfully under the suggested technique stated in this journal paper. The Table 2 info includes.

**Table 2:** Represents hardware and software components.

Component	Specification	Justification
Cameras	2 x 1/2.3“Sony Exmor R CMOS Sensors with Global Shutter, 2048x1536 pixels per sensor, 2 x 3.8 mm High-Quality Lenses, 66° HFoV, 2.67 mm Fisheye Lens, USB 3.0	Ensures distortion-free, high-resolution image capture with a wide field of view, crucial for accurate perception and scene reconstruction.
LiDARs	360° Field of View, 100 Hz Scan Rate, 150m Range, 0.1° Resolution, 4 scanning planes, comprehensive 360° coverage	Enables precise 3D mapping and object detection in dynamic environments with enhanced accuracy.
GPS	NovAtel SPAN-CPT ALIGN inertial and GPS navigation system, 9 axes, 100 Hz, GPS/GLONASS, triple antenna with Bluetooth and WiFi	Provides high-frequency, real-time localization for accurate positioning and navigation.
Python	Python 3.12.0	Chosen for its extensive libraries and ease of use in development, testing, and result analysis.
Deep Learning	Machine learning framework for perception and decision-making	Facilitates real-time object detection, image segmentation, and AI-driven decision-making.
Fully Convolutional Neural Network	Specific deep learning architecture for image processing and object detection	Optimized for high-performance image analysis, improving recognition and classification tasks.
VS Code	Update 1.85; Text editor and IDE	Offers a robust and customizable coding environment, enhancing productivity.
Anaconda Navigator	Anaconda Distribution 2023.03 installer, Python 3.10 base, supports 3.8 & 3.9, updated with Numpy, SciPy, Pandas, Matplotlib	Provides an efficient package manager and development platform for AI/ML projects.
Figma	Design and prototyping tool	Enables UI/UX design, ensuring an intuitive user experience for applications.

### 3.2. Datasets used

To generate a model for the proposed system, input from the following datasets was required.

- nuScenes Dataset: The nuScenes dataset is recognized as a leading open-source resource for autonomous driving. Gathered in Boston and Singapore, it utilizes an extensive sensor array, including a 32-beam LiDAR, six 360° cameras, and radars. With a vast repository of over 1.44 million camera images, the dataset encapsulates a wide spectrum of traffic scenarios, driving maneuvers, and unanticipated behaviours [27].
- LeddarTech PixSet Dataset: One of the newest open-source datasets created for research and development in autonomous driving is the Leddar PixSet. It is an exhaustive collection of data that has been captured using every sensor available in autonomous cars, such as radar, IMU, LiDARs, and cameras. The dataset contains full-waveform data captured from the Leddar Pixel, an original type of 3D solid-state flash LiDAR sensor. Over 1.3 million annotated 3D boxes can be noticed with a collection of 29,000 frames distributed over 97 sequences to enhance the usefulness for a variety of autonomous driving solutions [28].

### 3.3. Proposed model workflow

**Fig 2:**
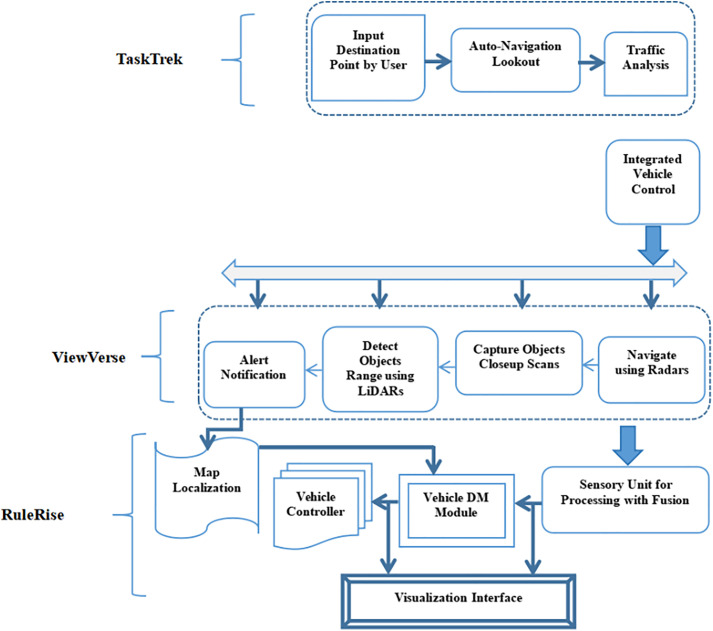
Proposed model framework flow design for autonomous vehicles.

The proposed architectural framework model for autonomous vehicle’s notion is illustrated in Fig 2 and it forms three separate parts:

- TaskTrek - It streamlines the vehicle’s braking response by concentrating on task-oriented features, including destination input, auto-navigation lookout, and traffic analysis to optimize vehicle control.
- ViewVerse - It captures perceptual aspects and utilizes sensor technologies like LiDARs and radars to comprehend the environment in real-time, improving the system’s precision through object detection and alert mechanisms.
- RuleRise -It controls rule-based decision-making, ensuring that braking actions conform to safety standards, legal regulations, and evolving driving conditions. It integrates map localization, vehicle controllers, and decision-making modules to process real-time sensory data for optimal vehicle responses.

In the TaskTrek component, the functionality include a touch-screen user interface with voice commands provides easy access to the system’s intuitive layout. It corrects input errors for correctness, evaluates routes accurately, and links them with mapping data. The Auto-Navigation Lookout takes control as the car moves on, using sensor data and smart mapping to map the path and identify threats. Relying on GPS, cameras, and LiDAR, Map Localization pinpoints the car’s exact location on the map and tracks its position in its lane. The car’s path is modified by driver assist systems, which are always aware of the traffic dynamics. Persistent and flexible, navigation autonomy maintains responsiveness through precise traffic monitoring and accurate localization.

The functionality of the ViewVerse component encompasses is To sustain vehicle effectiveness, Integrated Vehicle Control sends signals to actuators to control acceleration, braking, and steering. The steering control maintains lane alignment while brake assist handles the stop and avoids collisions. It interprets and transmits directions and maps out predetermined paths with GPS systems. Radar-assisted navigation uses ADAS radar sensors to monitor conditions and identify objects. Adaptive cruise control ensures safer following distances by modifying speed in response to radar data. Radar-based blind spot detection increases security by warning drivers of impending cars. When deployed in conjunction, these qualities offer precise control and comprehensive navigation, enhancing assurance and agility.

With its high-resolution cameras, the RuleRise component improves a vehicle’s awareness of surrounding elements such as traffic signs and road markers, guaranteeing accurate detection and adherence. Such evaluation helps the system make decisions, such as alerting the driver or adjusting the steering and brakes. Through laser pulse reflection monitoring, LiDAR technology creates three-dimensional maps of the surroundings, thus enabling effective travel for self-driving vehicles. While assessing traffic patterns, recommending alternate routes, and even keeping tabs on fuel levels, the Alert Notification System improves passenger comfort and security. Driving will become safer and more intelligent as a result of these technologies uniting.

Taken together, different viewpoints in RuleRise form a powerful, highly complex, and intelligent system.

**Sensory Unit for Processing with Fusion:** Data collected by multiple installed sensors, including cameras, radar, LiDAR, and ultrasonic sensors, is continuously fused by the essential sensory unit, which is in charge of data fusion, to create a seamless illustration of the outside world. With its thorough scene analysis and target identification capabilities, the device can identify various elements in the surrounding environment, including cars, bikes, pedestrians, and traffic signs, providing the detailed knowledge necessary for safe driving. Beyond simple data integration, the predictive algorithms of the unit use preceding and current data to forecast possible actions of identified objects, like car lane changes, pedestrian crossings, or bicycle turns. The device updates in real-time whenever the vehicle is in motion, ensuring quick reactions to changing circumstances and prospective threats. It also provides key collision and safety aids for the vehicle.

**Fig 3:**
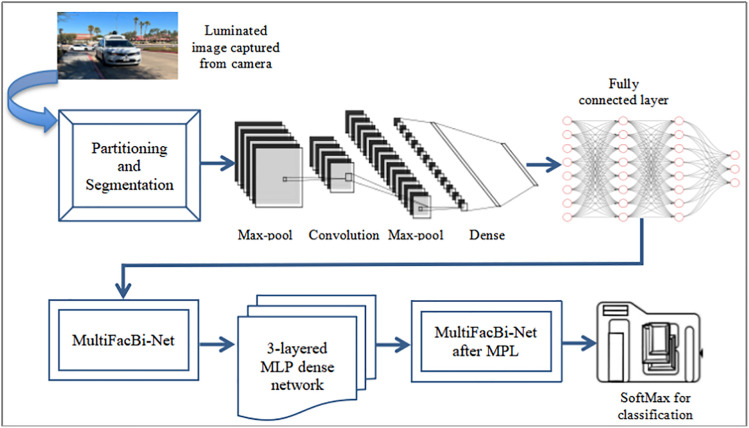
Components of AttentionDriveNet Model for Obstacle Detection.

The model utilizes the AttentionDriveNet technique, as in Fig 3, to consider input from vehicle sensory units. Leveraging a multifaceted approach and modern techniques, the deep learning model for image detection and categorization in self-driving vehicles analyzes visual input accurately and quickly. The preprocessing stage starts with converting the input images collected by the onboard cameras to grayscale to improve the system’s computational efficiency and robustness to changes in illumination. The visuals are partitioned using nears that yield semantically meaningful portions. Also, additional partitioning is done to allow grid-based sub-division to locally extract features. The computational heart of the algorithm is a fully convolutional neural network designed mainly for image computing applications. Features are extracted from the combination of kernels in the layers of the FCNN architecture over input characteristic maps; this, therefore, produces hierarchical representations of the imagery. Max-pooling techniques effectively down-sample the feature maps after these convolutional layers, preserving key geography while lowering figuring complexity. Afterward, the attribute maps are routed through dense layers and deflated to enable complicated feature interactions and non-linear transformations. Rectified Linear Units (ReLU) is an example of an activation function that promotes non-linearity and improves the model’s ability to identify intricate structures in the data. To speed up convergence and stabilize learning, batch normalization is applied. The model uses a multifaceted attention process to fixate on significant regions of the image while squelching obstacles. It improves the extraction of discriminative characteristics across several levels of abstraction, increasing its symbolic strength by continually allocating attention and building multi-scale attention maps.

Following attention-based processing, multi-perceptron modeling is used to refine the features further. A multi-layer perceptron, which has numerous hidden layers, transforms the feature vectors further. This allows the model to learn delicate, non-linear linkages between the source images and target classes. Weight decay and dropout regularization strategies are used in eq-1 to reduce overfitting and enhance generalization performance.


$$\begin{array}{c} y\;=\;f(\sum_{i=\text{0}}^{n}\left(W_{i}.X_{i}\;+\;b)\right) \end{array}$$ (1)

*y* is the output of the neuron.

*f* is the activation function.

*(W*_*i*_.*X*_*i *_*+ b)* represents the weighted sum of the neuron’s inputs.

*X*_*i*_ are the weights associated with each input X_i_.

*b* is the bias term.

In order to verify that the model gathers all the pertinent features for delicate categorization, the output from the multi-perceptron models is then absorbed by the multifaceted focus mechanism for iterative attentional analysis. Ultimately, a softmax layer that calculates the likelihood of each class outputs the classification of the model’s data.

An ability to combine modern methods of region forecasting, deep learning, and loss evaluation makes the model agile in handling all kinds of difficulties in image analysis and project management.

- **Vehicle DM Module:** In order to facilitate flawless system-to-system communication for speedy control and decision-making, the Vehicle Data Management Module would serve as a core module for controlling different data kinds within the vehicle, such as sensor and navigation data. It serves as a central hub for effective data transfer between various car systems, promoting cohesion and timely updates in actual-life circumstances. It is a regulatory element for independent tasks and maintains control until it resumes normal function, even in the event of a sensor inputting issue.
- **Vehicle Controller:** The vehicle controller, which actively monitors and regulates automotive actuators as the brakes, steering, and acceleration to guarantee a vehicle’s secure and comfortable ride, is the brains behind our system. The proposed model uses this unit to recognize roads with or without curves and potholes, challenging on-traffic scenarios etc embedding accurate and suitable sensors. With the help of traction unit and anti-locking brake, it regularly tracks vital operations like acceleration and steering to facilitate faster response to any possible deviations. Synchronized control occurs when this unit collects sensory data to properly regulate tasks across vehicle.
- **Visualization Interface:** This platform provides drivers robust information regarding the most promising route they can take. Vital information like nearby turns or alternate paths data are provided which adds the intelligence element in their decision making. Also, this module utilizes GPS and speed information to record vehicular speed and its exact location on road which enables the vehicle for proper navigation of its surrounding thereby ensuring safe driving. Besides, it notifies the driver about lane markers, traffic and road signals so that vehicle does not deviate much from its path.

**Pseudocode 1:**

*Step 1: Input – Source Image*

*source_image = input_source_image()*

*Step 2: Preprocessing – Convert to Grayscale*

*grayscale_image = convert_to_grayscale(source_image)*

*Step 3: Segmentation and Partitioning*

*segmented_image = segment_image(grayscale_image)*

*partitioned_image = partition_image(segmented_image)*

*Step 4: Convolutional Feature Extraction*

*convolutional_output = [ ]*

*for partition in partitioned_image:*

*feature_maps = [ ]*

*for convolutional_layer in convolutional_layers:*

*feature_maps.append(convolve(partition, convolutional_layer))*

*convolutional_output.append(feature_maps)*

*Step 5: Pooling and Non-linearity*

*pooled_output = [ ]*

*for feature_maps in convolutional_output:*

*pooled_maps = [ ]*

*for feature_map in feature_maps:*

*pooled_map = max_pool(feature_map)*

*pooled_maps.append(pooled_map)*

*pooled_output.append(pooled_maps)*

*Step 6: Flatten and Dense Layers*

*flattened_output = [ ]*

*for pooled_maps in pooled_output:*

*flattened_maps = [ ]*

*for pooled_map in pooled_maps:*

*flattened_map = flatten(pooled_map)*

*flattened_maps.append(flattened_map)*

*flattened_output.append(flattened_maps)*

*dense_output = [ ]*

*for flattened_maps in flattened_output:*

*dense_maps = [ ]*

*for flattened_map in flattened_maps:*

*dense_map = dense(flattened_map, weights, biases) # Using MLP formula*

*dense_maps.append(dense_map)*

*dense_output.append(dense_maps)*

*Step 7: Activation and Batch Normalization*

*activated_output = [ ]*

*for dense_maps in dense_output:*

*activated_maps = [ ]*

*for dense_map in dense_maps:*

*activated_map = activate(dense_map, activation_function)*

*activated_maps.append(activated_map)*

*activated_output.append(activated_maps)*

*normalized_output = [ ]*

*for activated_maps in activated_output:*

*normalized_maps = [ ]*

*for activated_map in activated_maps:*

*normalized_map = batch_normalize(activated_map)*

*normalized_maps.append(normalized_map)*

*normalized_output.append(normalized_maps)*

*Step 8: Multi-faceted Attention Mechanism*

*attention_output = multi_faceted_attention(normalized_output)*

*Step 9: Multi-Perceptron Modeling*

*perceptron_output = [ ]*

*for attention_map in attention_output:*

*perceptron_map = multi_perceptron_model(attention_map)*

*perceptron_output.append(perceptron_map)*

*Step 10: Softmax Classification*

*classification_output = softmax(perceptron_output)*

*Step 11: Output – Analyzed Image Data*

*an*alyzed_image_data = classification_output

To put it very briefly pseudocode 1 provides a well-organized image analysis engine that is prepared for the intricate demands of autonomous cars. At start, the captured images are filtered applying functional libraries so as to enhance its clarity. At start, the captured images are filtered applying functional libraries so as to enhance its clarity. The images are segmented and grayscale conversion is used for preprocessing. Simultaneous processing of those segmentation areas is done with Convolutional Neural Networks and these are structured to retrieve distinct features. It is followed by pooling which is integrated with random conversion to fetch significant variables within the model which optimizes its ability to recognize pattern accurately. The neural network is suitably utilized with proper bias with the traversal of condensed feature maps upon the layers. This procedure highlights the complicated random association among desired labels and input features. The combination of activation functions and batch normalization produces non-linearity, helping in convergent stability. The depth of feature representation increases by the use of a broad attention mechanism to flexibly center the inspection by the model on important regions in source data. Classification of the model’s output is done as softmax after attention-driven tuning; this Bayesian classification considers each class. The function details used in pseudocode is explained in Table 3. The pseudocode is a complex mix of some of the hottest recent techniques, including convolutional neural networks, multiple layer perceptrons, attention mechanisms, and softmax classifiers, in an attempt to deliver reliable and authentic visual analysis of data for autonomous driving needs.

**Table 3:** Functions details used in the pseudocode.

Function	Nuances
**input_source_image()**	Retrieves the initial snapshot that was snapped by the onboard cameras and is used as a baseline for more processing.
**convert_to_grayscale()**	Converts the input color image to grayscale, enhancing computational efficiency and simplifying subsequent processing steps.
**segment_image()**	Segments the grayscale image into meaningful regions or segments, facilitating the extraction of relevant features for analysis.
**partition_image()**	Further partitions the segmented image into smaller, manageable sections, aiding in localized feature extraction and analysis.
**convolve(image, kernel)**	Applies a convolution operation on the input image using a specified convolutional layer, extracting features and detecting patterns.
**max_pool(feature_map)**	Performs max pooling operation on the input feature map, reducing spatial dimensions while preserving important information.
**flatten(feature_map)**	Flattens the pooled feature map into a one-dimensional array, preparing it for processing by dense layers.
**dense(vector, weights, biases)**	Applies the multi-layer perceptron (MLP) formula to compute the weighted sum of inputs with corresponding weights and biases, introducing non-linear transformations.
**activate(vector, activation_function)**	Applies an activation function to the output of dense layers, introducing non-linearity to capture complex patterns and relationships.
**batch_normalize(vector)**	Normalizes the output of activation functions to stabilize training and improve convergence, enhancing the model’s robustness and generalization ability.
**multi_faceted_attention(data)**	Incorporates a multi-faceted attention mechanism to dynamically allocate attention to significant regions of the input, enhancing feature representation and focus.
**multi_perceptron_model(data)**	Utilizes a multi-layer perceptron (MLP) for further refining feature representations through multiple layers of perceptrons, capturing intricate, non-linear associations.
**softmax(data)**	Applies the softmax function to the output of the multi-perceptron model, computing the probabilities of each class and facilitating classification and decision-making.

## 4. Implementation results and analysis

It is of paramount importance to summarize the testing or data analysis findings at the results analysis section of the written piece. This section provides essential details for understanding these findings and how they could affect the development of autonomous car tech. Highlighting essential indicators of performance, critical metrics, or remarkable patterns found during the study gives readers an outline of the insights that will be further discussed in the next sections.

The Table 4 of contents that is displayed gives a brief synopsis of various motion planning strategies, outlining their unique approaches and benefits. Each strategy has distinct advantages and is suited to certain circumstances. Conventional methods, including graph search and optimization, provide assurances and accurate management, whereas sampling-based approaches outperform in high-dimensional environments and interactive applications. Path smoothness is given priority by curve interpolation, and deep learning that is, Fully Convolutional Networks and Segmentation Networks—shows promise in reproducing expert demonstrations, especially at the pixel level. The table highlights how the deep learning method can be used to achieve better results in motion planning duties.

**Table 4:** Graph techniques used for GPS localization and mapping.

Approach	Strategy	Benefit
Graph Search	Dijkstra’s [29]	Ensures optimal solution finding
	State lattice [30]	
Sampling-based	RPP (Rapidly-exploring Random Trees) [31]	Swift processing, adept in high-dimensional spaces
	RRT (Rapidly-Exploring Random Trees) [32]	
	RRT* (Rapidly-exploring Random Trees with bias towards the goal) [33]	
	PRM (Probabilistic Roadmap Method) [34]	
Curve Interpolation	Clothoids [35]	Produces seamlessly smooth paths
	Polynomials [36]	
Numerical Optimization	Newton’s Method [37]	Capable of deriving high-quality paths
	Numerical Non-linear Optimization [38]	
Deep Learning	AttentionDriveNet	Exhibits superior pixel-level imitation performance

The proficiency percentages in Table 5 are derived from extensive real-world testing and simulation-based evaluations under varying environmental conditions. These values are benchmark against industry standards and validated using datasets from autonomous driving research initiatives. Additionally, factors such as sensor resolution, range, and response time contribute to the assigned proficiency scores. With 95.3% proficiency, 3D LiDAR excels in precision and a variety of situations. Cameras have an efficiency of 73.81%, demonstrating their competence in recognizing objects. Radar is useful in bad weather because of its 81.72% efficiency. Important data points are provided by auxiliary sensors: odometer is at 39.8%, IMU is at 52.8%, and ultrasonic sensors is at 67.2%. GNSS at 42.9% and predictive modeling at 31.2% are areas with room for improvement, highlighting the continuous need for advancements in navigation and future prediction skills.

**Table 5:** Sensor suites for image detection in autonomous vehicles.

Sensor Suite	Scope in image detection and classification in Autonomous Vehicles	Proficiency
AttentionDriveNet	Highly accurate and effective for detecting objects in a wide range of environments, including those with low visibility.	95.3%
Radar (Radio Detection and Ranging) [39]	Less accurate than LiDAR, but effective for detecting objects in poor weather conditions.	81.7%
Camera [40]	Effective for identifying objects and their characteristics, such as their size, shape, and color.	73.8%
Ultrasonic sensors [41]	Sensors that emit sound waves and measure the time it takes for them to reflect back.	67.2%
Inertial measurement unit (IMU) [42]	A sensor that measures the vehicle’s orientation and acceleration.	52.8%
Global Navigation Satellite System (GNSS) [43]	A system that provides the vehicle’s position and location.	42.9%
Odometry [44]	A method of estimating the vehicle’s position and movement by measuring the rotation of its wheels.	39.8%
Predictive modeling [45]	A method of using historical data and machine learning to predict the future behavior of objects in the environment.	31.2%

**Fig 4:**
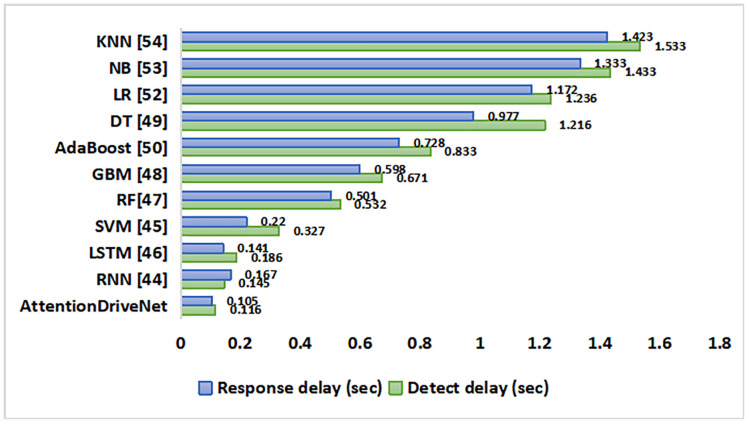
Latency component analysis for various predictive learning methods.

The Fig 4 showcases the results of this thorough investigation regarding environmental component detection and response methodologies, which highlights noticeable patterns. With their remarkable detection and response times (0.116 and 0.105 seconds, respectively), the suggested Fully Convolutional Neural Networks are lightning fast and a great option for real-time applications. Although they are a little slower, recursive neural networks and long short-term memory (LSTM) networks showed promise when processing sequential input, which makes them useful for tasks requiring movement or environments with a lot of variation. However, although yielding good accuracy, classic ensemble approaches such as Random Forests and Gradient Boosting Machines are slower. Additionally, the slowest response times more than a second are displayed by simpler models like Naive Bayes, Decision Trees, Logistic Regression, and Linear Regression, indicating their limitations for real-time applications. This detailed analysis highlights the trade-offs between response speed and processing efficiency present with different detection and response techniques.

The image classification system of the autonomous car shows consistent operation with different objects and views in Table 6. From every angle, pedestrians may be identified with the highest accuracy when regarded from the front. Cars and trucks in particular show excellent accuracy in both the right and left views. Motorbikes and bicycles exhibit strong performance from every angle. Excellent precision is displayed by traffic signs and lights, especially in the Front and Back points of view. Potholes, animals, and crosswalks are all clearly marked, with optimal views for each. Trees are quite accurate; the Front view is the most authentic. Overall, the determined mean obstacle detection accuracy from right, left, front and back views are 88.3%, 83.8%, 91.4% and 89.9% respectively. All things considered, the system performs outstandingly in subtle finding of objects, which adds to its complete knowledge of the surroundings for safe autonomous navigation.

**Table 6:** Objects classification accuracy of the model from different camera angles.

Object	Right View (%)	Left View (%)	Front View (%)	Back View (%)
**Pedestrians**	93.5	87.8	96.2	93.6
**Vehicles**	94.1	92.6	90.7	89.6
**Bicycles**	90.4	85.8	94.2	94.6
**Motorcycles**	96.3	91.8	96.8	97.6
**Traffic Signs**	81.7	79.3	80.5	79.2
**Traffic Lights**	80.4	81.2	86.4	87.8
**Animals**	81.7	80.9	88.7	90.6
**Potholes**	95.7	80.8	96.6	83.5
**Trees**	82.6	78.4	93.3	91.8
**Crosswalks**	86.2	79.5	90.6	89.5

The Table 7 presents a comparison of various object detection models, including Hybrid YOLOv3, Hybrid SSD, Hybrid CenterNet, Hybrid RetinaNet, YOLOv5, and AttentionDriveNet. Among these models, AttentionDriveNet stands out with the highest accuracy of 95.3%. This superior accuracy is coupled with a relatively low processing time of 0.36 seconds, making AttentionDriveNet an efficient choice for real-time applications. Moreover, it maintains a commendable recall rate of 91.7%, ensuring robust performance in detecting relevant objects. Overall, AttentionDriveNet emerges as a top-performing model in terms of accuracy, processing speed, and recall, showcasing its effectiveness in object detection tasks.

**Table 7:** Performance metrics comparison analysis of image classification models.

Model	Detection accuracy (%)	Processing time (in sec)	Recall (%)
Hybrid YOLOv3 [17]	73.217	0.33	78.3
Hybrid SSD [18]	89.871	0.38	83.3
Hybrid CenterNet [19]	84.72	0.47	83.1
Hybrid RetinaNet [20]	90.161	0.34	88.6
YOLOv5 [21]	90.518	0.48	89.9
AttentionDriveNet	95.3	0.36	91.7

**Fig 5:**
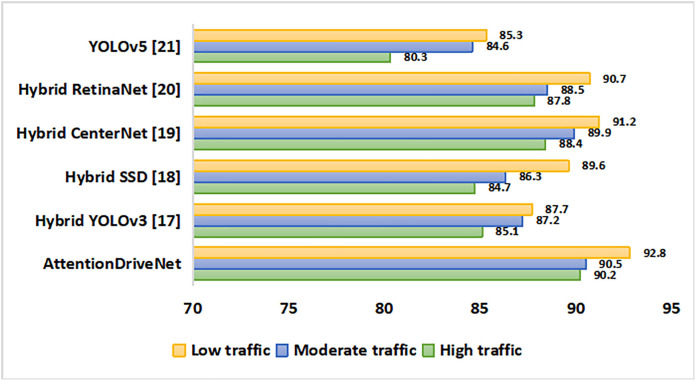
Comparison of image classification models in heavy and light traffic.

Using a comprehensive analysis that includes a range of on-road scenarios, we compare the performance of five different models in terms of appropriate categorization and image recognition. In context to the traffic load on roads, the model is validated. The traffic is classified into high, moderate and low types as shown in Fig 5. In different traffic loads, the model performed very well. 92.8% object detection accuracy was seen during low traffic while during high traffic, the accuracy slightly dips to 90.2%. Among the compared models, Yolov5 noted a lesser prediction accuracy.

**Fig 6:**
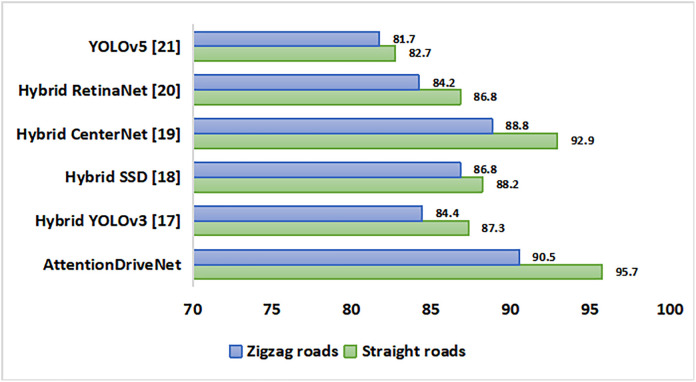
Comparison of image classification models in Straight and Zigzag roads.

**Fig 7:**
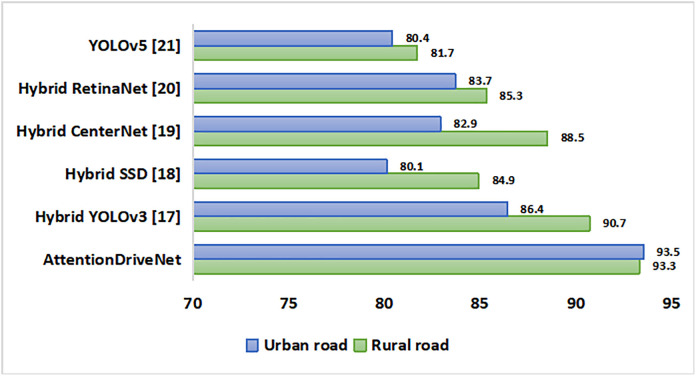
Comparison of image classification models in rural and urban roads.

Further the roads can be either straight direct type or zigzag with multiple turns. Upon evaluation, it was observed that the developed model was able to identify objects with optimum accuracy in both kinds of roads. The accuracy shoots up to 95.7% in straight roads while it is relatively low in zigzag roads (90.5%) due to presence of turns. Hybrid CenterNet model comes close to our model in predicting objects accurately. Fig 6 shows the overall analysis. The model’s performance is also checked in both rural and urban regions as depicted in Fig 7. Interestingly it recorded similar object prediction rate with 93.3% in rural zone and 93.5% in urban areas. The outcome is quite good as compared to other predictive models.

**Fig 8:**
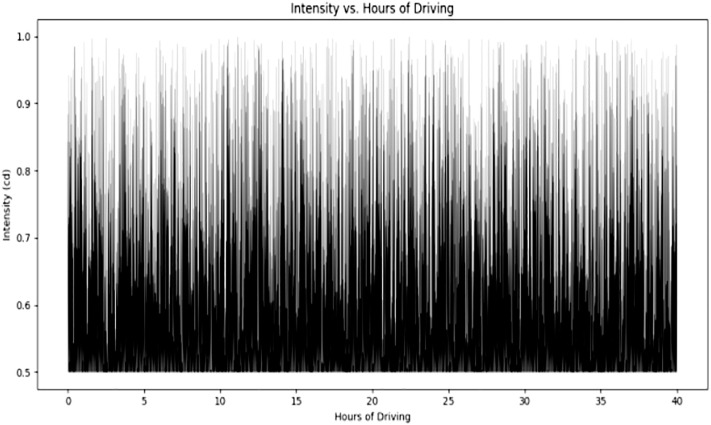
Variation of Intensity of objects with driving hours.

Fig 8 illustrates the relationship between the intensity of objects and the duration of driving. Over the course of a nearly 39-hour experiment, we observed which objects exhibited the highest intensity, enabling driver-less vehicles to adapt their behavior based on the proximity of objects. The average intensity was calculated to be 0.409 Cd. This threshold distinguishes between low-intensity objects or distant objects.

**Fig 9:**
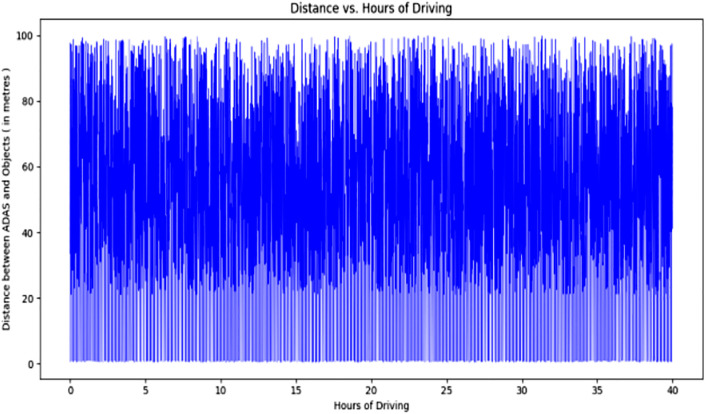
Relationship between distance from objects and driving hours.

Fig 9 illustrates the relationship between the distance from objects and hours of driving. After conducting a nearly 39-hour driving experiment, we observed that objects closer to the car tend to cluster in the lower regions of the graph, indicating their proximity in bulk. Conversely, objects represented by points at higher notes on the graph are more distantly located, and their distances are depicted accordingly.

**Fig 10:**
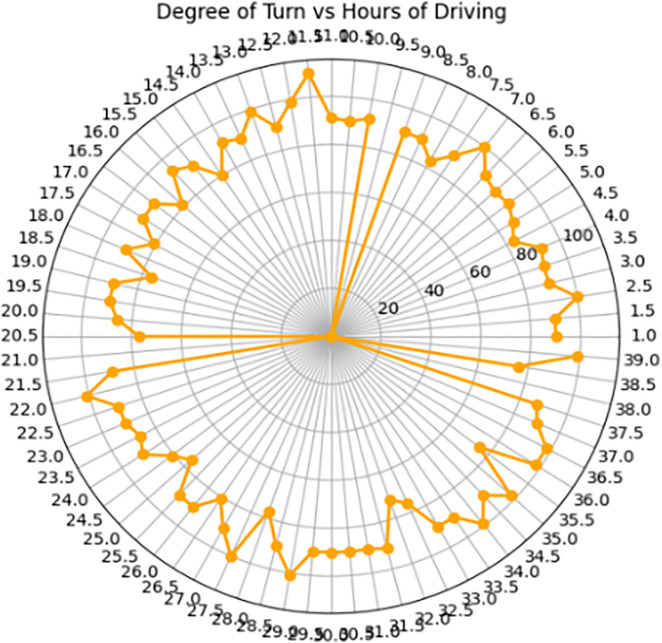
Degree of turn with driving hours analysis.

In the provided radar chart, Fig 10 shows the relationship between the degree of turn and the number of hours of driving over a sample of 39 hours. The default degree of 90 represents the vehicle traveling in a straight line. As the degree decreases from 90, it indicates a left turn, with the magnitude determining the sharpness of the left turn. If the degree surpasses 90, it signifies a right turn. The graph exhibits several instances where the points return to the origin, indicating U-turns. These occurrences suggest instances where the vehicle reverses its direction during the journey. The orange line in the radar chart visually represents the variation in the degree of turn throughout the driving duration, providing insights into the dynamics of the vehicle’s movements during the sampled hours.

### 4.1. Discussion

To improve autonomous vehicles’ brake reaction, selection precision, and real-time navigation, the proposed solution combines TaskTrek, ViewVerse, and RuleRise. This paradigm maximizes vehicle productivity and security in volatile circumstances by fusing rule-based decision-making, task-oriented control, and perceptual awareness. Our hybrid method, in contrast to conventional rule-based or solely AI-driven models, strikes a compromise between regulatory devotion and ability to adapt, securing compatibility while improving navigation accuracy. The system also enhances adaptive navigation, lane identification, and obstacle detection by utilizing various sensor modalities, which makes it appropriate for intricate urban settings.

Although the model suggested performs well under a variety of circumstances, some restrictions reduce its overall durability. In severe weather situations where sensor accuracy is reduced, like intense rain, thick fog, or snow, the model may perform inadequately. The making of decisions may also be impeded by unstructured or highly volatile surroundings, such as crowded urban junctions or poorly defined country roads. The effectiveness of real-time navigation can also be impacted by alterations in lighting, sensor noise, and unforeseen obstructions like stray animals or pedestrians.

## 5. Conclusion

Our extensive testing confirms the effectiveness of our hybrid model in enhancing autonomous vehicle capabilities. AttentionDriveNet achieved the highest object detection accuracy (95.3%) with a rapid processing time of 0.36 seconds, ensuring real-time efficiency. It demonstrated strong performance across varying road conditions, with 95.7% accuracy on straight roads and 90.5% on zigzag roads. Object classification accuracy varied by viewing angle, with obstacle detection averaging 91.4% (front view), 89.9% (rear view), 88.3% (right view), and 83.8% (left view), ensuring comprehensive environmental perception. Sensor analysis revealed 3D LiDAR as the most precise (95.3%), followed by Radar (81.72%) and Cameras (73.81%), while GNSS (42.9%) and predictive modeling (31.2%) require improvement. The model maintained high accuracy in different traffic conditions (92.8% in low-traffic, 90.2% in high-traffic). Distance analysis during a 39-hour driving experiment helped refine vehicle response, with an average object intensity threshold of 0.409 Cd. Radar-based turn detection further highlighted dynamic vehicle maneuvering, including U-turn recognition.

The research underscores the notable developments in autonomous vehicle technology, demonstrating their capacity to transform transportation networks, enhance security, and transform the driving confront. This study presents a novel navigation model for self-driving vehicles using advanced predictive intelligence and remote sensing. A key feature is smarter Mobility Fusion, which integrates cameras, radar, and LiDAR for improved vehicle-to-infrastructure interactions and pinpoint navigation. Tools like Google Maps are integrated for real-time analysis and dynamic path optimization to address traffic concerns. Increasing the amount of visual data recorded, 360-degree LiDAR, and high-resolution cameras improve object classification. Our flexible integration maximizes processing efficiency using Neural Networking with Attention for targeted object recognition. Intending to advance self-driving vehicle technologies, this model promises intelligent, adaptable, and dependable performance in various real-life situations.

Looking ahead, the scalability of this model can be further enhanced through seamless integration with emerging sensor technologies such as high-resolution LiDAR, next-generation radar systems, and AI-powered vision modules. Additionally, incorporating real-time data exchange with cloud and edge computing platforms can improve decision-making efficiency. From a regulatory standpoint, as autonomous vehicle legislation continues to evolve, adapting the model to align with emerging safety standards and compliance requirements will be crucial. Future developments may focus on ensuring compatibility with global regulatory frameworks, enabling widespread deployment across diverse transportation ecosystems.

## 6. Future scope of study

Even though the proposed framework shows notable gains in autonomous vehicle decision-making for brake reaction and navigation, there are a number of areas that could use more investigation and improvement:

- *Handling extreme weather conditions:* The model’s performance can be further tested and optimized for adverse weather conditions such as heavy rain, snowfall, and fog, where sensor accuracy may degrade. Advanced sensor fusion techniques and AI-driven weather adaptation models could enhance system reliability.
- *Adaptability to varied road types:* The current model is optimized for structured urban and highway environments. Further studies can focus on improving performance in unstructured terrains, off-road conditions, and highly congested city traffic. Incorporating high-definition mapping and reinforcement learning-based adaptive navigation can improve maneuverability.
- *Improvement in sensor integration and fusion:* With the continuous advancement in sensor technologies, future configurations will incorporate next-generation LiDAR, radar, and camera fusion techniques to enhance object detection and classification accuracy. Research can focus on minimizing sensor latency and leveraging AI-driven data synchronization for real-time, high-precision decision-making.
- *Security Threats and Countermeasures for UAVs:* Future studies should address security risks to UAVs, such as data injection, jamming, and GPS spoofing. Real-time danger reduction is possible with AI-driven detection, and secure, impenetrable communication is ensured with blockchain integration.
